# Prescribing Antidiabetic Medications Among GPs in Croatia—A Real-Life Cross-Sectional Study

**DOI:** 10.3390/biomedicines13061491

**Published:** 2025-06-17

**Authors:** Tomislav Kurevija, Ema Schönberger, Matea Matić Ličanin, Ines Bilić-Ćurčić, Ljiljana Trtica-Majnarić, Silvija Canecki-Varžić

**Affiliations:** 1 Faculty of Medicine, J.J. Strossmayer University of Osijek, 31000 Osijek, Croatia; tkurevija6@gmail.com (T.K.); ema.schonberger7@gmail.com (E.S.); matic.ma@gmail.com (M.M.L.); ibcurcic@mefos.hr (I.B.-Ć.); ljiljana.majnaric@gmail.com (L.T.-M.); 2 Health Center of Osijek-Baranja County, 31000 Osijek, Croatia; 3 University Hospital Center Osijek, 31000 Osijek, Croatia

**Keywords:** type 2 diabetes, cardiovascular risk, SGLT2ins, GLP-1 RAs, family medicine

## Abstract

**Background**: Advances in the treatment of type 2 diabetes (T2D) in recent decades have been primarily focused on its broader understanding in the context of the possibility of preventing the development and progression of the disease and of cardiovascular (CV) complications. Nevertheless, worldwide research indicates that individuals with T2D are still under-regulated, both in terms of glycemic control and in preventing CV complications. The aim of this study was to examine Croatian general practitioners (GPs)’ practice and patterns in prescribing antidiabetic medications and their understanding of guidelines. **Methods**: Research was conducted using a self-designed anonymous survey, which was delivered to the e-mail addresses of GPs throughout Croatia in digital format. Respondents were solely GPs, without any restrictions with regard to their characteristics. Data on the number of individuals diagnosed with T2D and prescribed a specific medication were based on declarations by respondents from their e-health records. **Results**: Approximately 59% of individuals with T2D are cared for solely by GPs. In terms of achieving targeted values of HbA1c, 47% of individuals with T2D are well regulated. Almost all the respondents claim that they review prescribed T2D therapy at least once a year. A total of 47.6% of respondents have read and entirely understood the EASD/ADA guidelines, but 58.3% apply the dual principles of controlling HbA1c levels and CV risk in the treatment of T2D. In individuals with associated CV comorbidity, SGLT2ins were the most frequently prescribed. **Conclusions**: The results indicate that Croatian GPs are still inclined to apply outdated paradigms of T2D treatment but that they are gradually accepting new regimens of care and recommendations for prescribing novel, more effective medications.

## 1. Introduction

The practice and attitudes of physicians in diagnosis and treatment, especially of chronic diseases, differ in various healthcare systems around the world. Although they follow the same guidelines, physicians’ decisions are vastly conditioned by the regulations of healthcare systems, while the diversity itself is affected by the differing organization of systems worldwide. Therefore, it is important to bear this in mind when studying papers on physicians’ habits and attitudes in disease treatment. The Croatian healthcare system is a public service divided into primary, secondary, and tertiary levels. The first, and often the only, treatment individuals with type 2 diabetes (T2D) receive is from their general practitioner (GP). Although the diagnosis and treatment of uncomplicated T2D cases is the domain of GPs, in practice, patients are also referred to a specialist diabetologist. Decisions about what proportion of individuals with T2D GPs decide to refer and at what point over the disease duration, as well as the presence of possible complications and associated cardiovascular (CV) risks, is dependent on the physician. This paper attempts to answer these questions.

A specialist diabetologist usually cares for complex T2D cases with associated comorbidities or complications as well as individuals with T1D, although GPs are also trained to introduce and evaluate insulin therapy. GPs in Croatia have a very broad role and considerable administrative burden. In general, in the context of patients with chronic diseases such as diabetes and CV diseases (CVDs), GPs monitor and evaluate their condition and, depending on the status and type of disease, periodically refer them to specialists for check-ups. Finally, in Croatia, any medical doctor can work as a GP, although in recent decades, the tendency of the system has been to refer GPs to specialize in family medicine (FM). During their specialist training FM residents work in teams with their mentors, who are senior and experienced FM specialists, and spend part of their training working in hospital departments most closely related to the scope of GP’s service.

Despite the wide range of antidiabetic medications currently available, less than 60% of patients achieve adequate glycemic control, with glycated hemoglobin (HbA1c) levels below 7% [1]. This is partly due to the high cost, inadequate patient education, and other factors that contribute to what is known as therapeutic inertia [2]. The term indicates the healthcare providers’ delay in initiating and intensifying pharmacologic treatment [3]. Many factors were identified as influencing therapeutic inertia, which can be classified as those related to the healthcare providers, the patients, and the healthcare system. The most important reason is considered to be the low adherence of GPs to the guidelines’ recommendations for pharmacological treatment, which may be due to the lack of GPs’ knowledge or to the guidelines’ shortcomings in providing support to the GPs in their decision making.

Although T2D treatment recommendations have been widely published, their use in general practice remains unsatisfactory. GPs’ characteristics, the healthcare system’s organization, and GPs’ engagement in specialized networks may all have an impact on guideline adherence [4]. In addition, the guideline manuals are usually too extensive and complex, making successful implementation difficult. Furthermore, they frequently fail to provide remedies for unique cases. Overall, clinical guidelines are often not intended to accommodate the working style of GPs and do not provide recommendations according to the specific characteristics of the patient. Namely, patients with T2D are very heterogeneous in terms of their physical (age, obesity, comorbidities, frailty, functional disorders), and psychosocial characteristics (socioeconomic status, personality traits, culture, living conditions, health literacy), and characteristics of the disease (T2D duration and the time of onset, the pace of progression, complications). The lack of competency-based workflows and efficient communication models with specialties may also be an important cause of GPs’ insufficient compliance with recommendations [4].

In recent years, pharmaceutical companies have aimed to develop new medication molecules with greater selectivity and specificity. For example, addressing key insulin-related dysfunctions in T2D can improve patient outcomes and lessen the disease’s clinical impact. Over the past two decades, the management of T2D has evolved from a strictly “glucocentric” focus to a more comprehensive approach that addresses medication-related side effects such as hypoglycemia and weight gain as well as the CV risk [5]. The latest “Standards of Care in Diabetes” from the American Diabetes Association (ADA) recommend a person-centered approach to medication selection for adults with T2D [6]. For individuals with an established or high risk of atherosclerotic cardiovascular disease (ASCVD), HF, or chronic kidney disease (CKD), treatment should prioritize agents that reduce the CV and renal risks. Sodium–glucose co-transporter 2 inhibitors (SGLT2ins) and/or glucagon-like peptide-1 receptor agonists (GLP-1 RAs) are recommended for glycemic control and CV risk reduction, regardless of HbA1c levels.

A meta-analysis demonstrated that SGLT2ins reduce HbA1c by about 0.7% compared with using a placebo [7], and individuals with T2D treated with SGLT2ins experience greater weight loss than those on a placebo [8]. Cardiovascular outcome trials (CVOTs) have shown that several SGLT2ins, including empagliflozin, dapagliflozin, and canagliflozin, provide superior CV benefits compared with the placebo in patients with T2D who either have or are at risk for ASCVD [9]. The EMPA-REG OUTCOME trial, for example, revealed significant reductions in both the CV risk and all-cause mortality in T2D patients with CV disease treated with empagliflozin [10]. A meta-analysis of five randomized controlled trials further confirmed that SGLT2ins lower the risk of CV death and hospitalization for HF across a wide range of HF patients, establishing them as essential therapy for HF regardless of the ejection fraction [11]. Moreover, post hoc analyses from CVOTs indicated kidney-protective effects resulting in the initiation of specific kidney outcome trials, which proved renal protection, reduced albuminuria, and slowed the progression of diabetic nephropathy [12].

GLP-1 RAs are another innovative class of antidiabetic medications that mimic the incretin hormone GLP-1. Beyond glycemic control and weight loss, GLP-1 RAs offer significant CV benefits, with clinical studies highlighting their role in reducing the risk of major CV events, including heart attack and stroke, without increasing hypoglycemia [13]. The CVOTs for dulaglutide, liraglutide, and once-weekly subcutaneous semaglutide consistently showed such a significant reduction in mortality and CV events that they are recommended in guidelines for high-risk patients [14]. Long-term studies confirm the efficacy and safety of GLP-1 RAs in glucose management, weight reduction, and CV protection, thus consolidating their role in diabetes treatment [15].

With the availability of a large number of antidiabetic medications and the rapid changes in the guidelines for the treatment of T2D, the prescription of these medications in a manner specific to an individual patient becomes a great challenge that easily leads the physician toward therapeutic inertia. This applies particularly to GPs who need to take into account the different characteristics of patients. In addition to assessing the presence of CV complications and CV risk and the degree of target organ damage, when planning medication therapy, GPs also consider the patient’s age, nutritional status, duration of their T2D, socio-economic status (availability of a particular medication), and side effects of the medication depending on the presence of comorbidities, as well as patient preferences [16]. Therefore, it is of particular importance to understand the way GPs plan medication treatment in light of new guidelines that give preference to medications with a CV benefit to T2D individuals with CV complications or a high CV risk. The aim of this research was to examine GPs’ knowledge of guidelines, patterns of medication therapy, attitudes about prescribing antidiabetic medications, and cooperation with endocrinologists in Croatia.

## 2. Methods

### 2.1. Study Design

This study was a quantitative survey research study with the target population being GPs across Croatia. To ensure equal representation from all regions of Croatia and based on previous research [17], we divided the respondents into six geographical regions based on similar sociological and economic characteristics as well as on health and dietary habits. These regions were named Western, Eastern, Northern, Southern, Central, and the city of Zagreb. The study was prepared following the STROBE checklist for cross-sectional studies [18]; a filled-in STROBE Statement is provided in Supplementary Table S7.

### 2.2. Description of the Instrument

The instrument of the study was a self-designed survey questionnaire created by a principal and a senior researcher based on experiences from clinical practice and the literature. Respondents (GPs) were reached via publicly available e-mail addresses of their practices and health centers. Aside from a link to the online survey, hosted on the Google Form platform, potential respondents were sent a cover letter with detailed information on the study design and purpose as well as instructions for the data collection and for answering the questions. Respondents approached the survey on a voluntary basis and completely anonymously, giving their informed consent to be made familiar with the study rules.

Most of the survey questions related to the practice, knowledge, and experience of the respondents, while a certain number of questions required exact data about individuals with T2D, which were obtained from the e-health records of the patients. Despite the impossibility of carrying out official external verification for the purpose of quality assurance, before accessing the questionnaire, respondents signed an informed consent form in which they declared entering accurate data. In this regard, we also have to declare a certain limitation that arose. As e-health records of Croatian GPs are not specifically intended for data research, we only made it a requirement to have the types of data we knew were present in e-health records (such as demographic data, comorbidities, and prescribed medications). For the foregoing reasons, we did not apply any standard report recommendations for using data from e-health records, such as, e.g., the RECORD checklist [19].

The self-design survey questionnaire that served as the instrument of this study consisted of two thematically related segments integrated into one online document. The first segment of the survey, aside from examining information on the respondents’ general characteristics, investigated the current practice of GPs in prescribing antidiabetic medications. The second segment of the survey questionnaire provided insights into the barriers that GPs encounter in the process of prescribing novel antidiabetic medications, as well as potential methods for the optimization of their prescription. In terms of the question form, this included closed-ended questions, including both single-selection and multiple-selection questions, while some questions contained a 5-point Likert Scale. The 5-point Likert scale was used in multiple questions with various meanings, mostly to express the level of importance, priority, or agreement with a specific issue. In all variants, a Likert grade of 1 indicates a low level, while a grade of 5 indicates a high level. The meaning of an individual Likert scale is detailed in the caption of each figure/table or associated text.

In this study, although some results from both segments of the survey are represented, our primary observations are from the results of the first segment, which focus on the practice of prescribing antidiabetic medications and which are expressed using descriptive statistics. In our previous publication, we presented the results based mainly on the second segment of the survey questionnaire, which examined barriers to prescribing antidiabetic medications and their correlation with the level of self-confidence of GPs when prescribing them on their own initiative, without specialist recommendation [20].

### 2.3. Validation Procedures and Sample Representativeness

In terms of validating the instrument, we conducted all the necessary procedures that are required for this type of survey research. After the initial design of the instrument, a few independent experts in the field of diabetology and FM, two university professors and two assistants (PhD students), revised the designed survey questionnaire and provided comments based on which, after joint debate, minor modifications were made, and the final version was defined. Then we conducted the six-month pilot phase (from October 2023 to March 2024), which included 86 respondents with 12,000 individuals with T2D under their care.

Based on the results of the pilot phase, we tested the respondent response and data relevance and performed early data processing to estimate the internal consistency of the data related to GPs’ attitudes. Cronbach’s α was 0.78. Furthermore, at the end of the survey, we repeated similar validity procedures for the data representing numbers (facts) and attitudes. The Cronbach’s α for the whole sample was 0.76.

We calculated the sample size in terms of the aforementioned broader research demands. To detect a significant mean difference between continuous variables in two independent groups (high and low level of self-confidence in medication prescription) with a medium effect size (d = 0.5), a required sample size of 128 respondents was estimated, assuming a significance level of 0.05 and a statistical power of 0.8. Furthermore, for bivariate and multivariate regression analysis, the results of which were presented in our previous paper [20], a minimum of 10 respondents per predictor was required. Consequently, the total minimum sample size was determined to be 180 respondents. Considering that this study aimed to examine GPs from the entire Croatian region, we decided to conduct the research in digital form (online).

**Figure 1:**
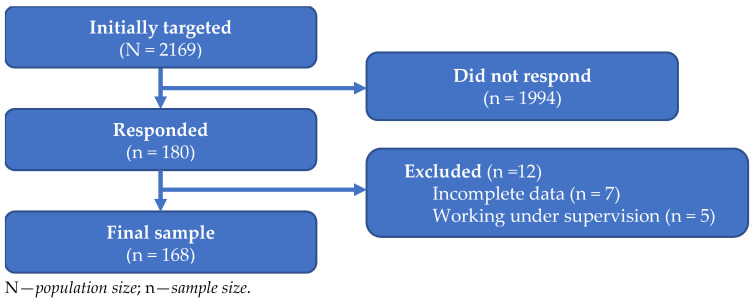
Data flow diagram representing respondents’ engagement in the study.

Respondents’ engagement in the study was represented by the data flow chart shown in Figure 1. The entire Croatian GP population initially targeted was reached via publicly available e-mail addresses of practices. We continued to seek responses from all the potential participants multiple times during a second phase from June to November 2024, when we finished the research study due to a rapid change in the research field, as well as due to a declining interest among potential respondents. Among 180 participants, 12 were excluded due to incomplete data and because they worked under a supervisor (i.e., non-independently), which formed the final sample of 168 respondents included in the analyses.

**Figure 2:**
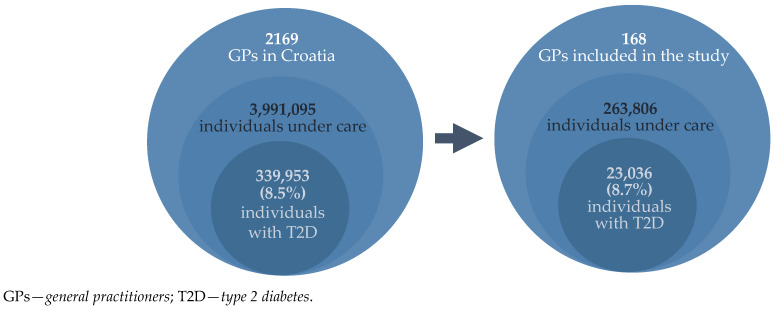
Relationship between national health data and the obtained results.

To ensure that a slightly smaller sample than calculated (<10%) would not affect the validity of the research, we tested the representativeness of the sample, comparing obtained results of the general characteristics of respondents and their patients with national health data. The obtained sample met the representativeness criterion, confirming an equal participation by GPs from all counties in Croatia (Supplementary Table S1), as well as by GPs of both younger (<45) and older (≥45) ages, and gender ratio (around two thirds are women) was also representative [21]. Furthermore, the portion of FM specialists in our research was 49.4% vs. 50.4%, in accordance with data from the national Croatian Medical Chamber register, while the portion of FM residents was 17.3% vs. 14.6%, respectively [21] (Table 1). In regard to the characteristics of the respondents’ patients, the obtained data on the number of individuals with T2D (8.7%) is in accordance with the data from the national health register (8.5%) (Figure 2), as well as with the data on their age stratification [22].

Statistical analyses were performed using the MedCalc^®^ Statistical Software version 23.0.6 (MedCalc Software Ltd., Ostend, Belgium; https://www.medcalc.org; accessed on 26 November 2024). The study was prepared following guidelines for reporting research findings in biomedicine and health sciences [23].

## 3. Results

### 3.1. Respondents (GPs) and Insured Individuals Under Their Care

The total population of 2169 GPs in Croatia [21] have 3,991,095 insured individuals under their care [24]. According to annual data from the Croatian Institute of Public Health, there are 339,953 individuals diagnosed with T2D, which equates to 8.5% of the total Croatian population [22]. The national data correlate with our findings, where 168 GPs included in the study care for about 263,806 insured individuals, among which there are 23,036 diagnosed with T2D (8.7%) (Figure 2).

In some GPs’ practices, the number of insured individuals ranged from 500 to 2500, with a median number of 1594 (IQR 1315–1850), while the number of individuals with T2D among the practices varied from 15–370, with a median number of 133 (IQR 95–179). General characteristics of the respondents and the insured individuals under their care are similar to those in national health data records [21]. The majority of respondents were women (66.1%), FM specialists (49.4%), and working in the urban area (67.9%) (Table 1), with a median age of 45 years. The regional diversity of the respondents and of the individuals under their care is shown in Supplementary Table S1. The examined respondents had 23,036 individuals with T2D under their care, mostly aged 60 to 80 years (Table 2). In regard to associated CV comorbidities among the T2D individuals, the highest occurrences were of hypertension (58.6%) and coronary artery disease (CAD) (20.0%). Prescription rates of antidiabetic medications with cardio- and reno-protective effects were 35.0% for DPP-4ins, 20.9% for SGLT2ins, and 14.4% for GLP-1 RAs (Table 2).

### 3.2. Practice of Prescribing Antidiabetic Medications

**Figure 3:**
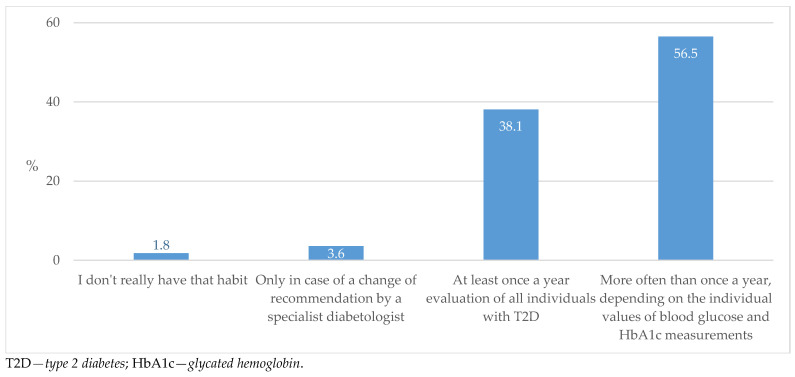
Therapy evaluation frequency in individuals with T2D.

Almost all of the respondents pointed out that they evaluate antidiabetic therapy at least once a year, with over half of them doing this even more frequently depending on the glycemic parameters (Figure 3).

**Figure 4:**
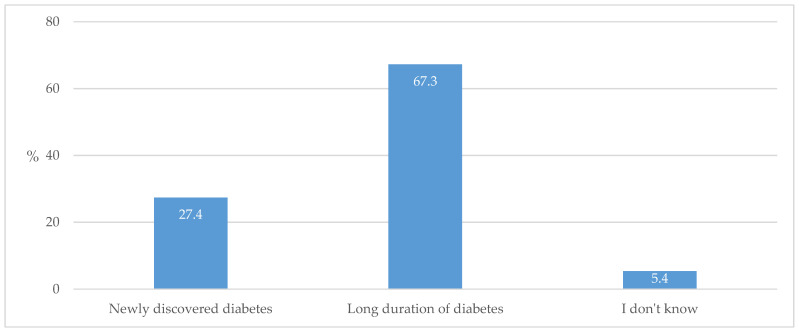
Individuals with T2D whose therapy is changed more frequently.

Furthermore, when asked about which individuals with T2D they change therapy for more frequently, two thirds of the respondents marked “those with long duration of the disease” (Figure 4).

Among the highest priority indications for prescribing GLP-1 Ras, the ones most selected by the respondents were obesity and the achievement of target values of HbA1c, while left heart ventricle hypertrophy (HLV) was the least. In the case of prescribing SGLT2ins, respondents primarily selected heart and kidney failure and the achievement of target values of HbA1c, while the least important indication was obesity. A detailed list of indications and their priority rating on the Likert scale for both of these medication groups are presented in the form of a heatmap in Table 3.

When asked the importance of the influence of the fear of the side effects in the process of prescribing, less than a quarter of the respondents found this issue to be a very important factor (Likert grades 4 and 5), with the lowest percentage in terms of prescribing DPP-4ins (Table 4).

**Figure 5:**
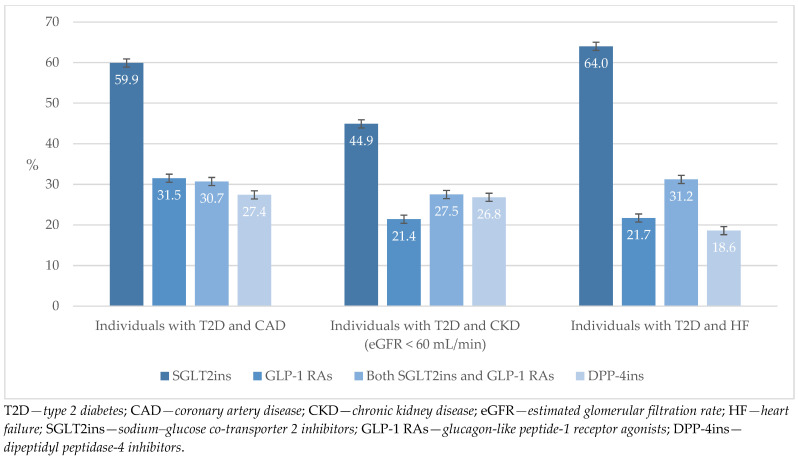
The most prescribed medications for individuals with T2D and associated comorbidities.

As we reported earlier in this section, there were a certain number of individuals with T2D and associated comorbidity; therefore, we asked the respondents to declare which antidiabetic medications were dominantly prescribed in such cases. The results indicate that by far the most frequent therapy option in such cases is SGLT2ins, followed by an equal frequency of prescribed GLP-1 Ras and combinations of SGLT2ins and GLP-1 RAs and DPP-4ins. Calculated values of the standard error of the mean (SEM) are also represented (Figure 5). The obtained SEM values indicate low variability in the sample and a good level of representativeness. A detailed list of all the therapy options, rated on the Likert scale, can be found in Appendix A, Table A1.

There are numerous factors that can influence the process of prescribing medications; therefore, we asked the respondents to mark the most important reasons that contribute to their decision when prescribing medications to individuals with T2D. A detailed list of the factors, rated in order of the importance of their influence, is shown in Supplementary Table S2. Furthermore, in Supplementary Table S3 we present several additional findings that relate to respondents’ attitudes and habits in the practice of prescribing antidiabetic medications, which provide close insights into their strategies in decision making in the process of treating T2D.

### 3.3. Referral to Specialists

**Figure 6:**
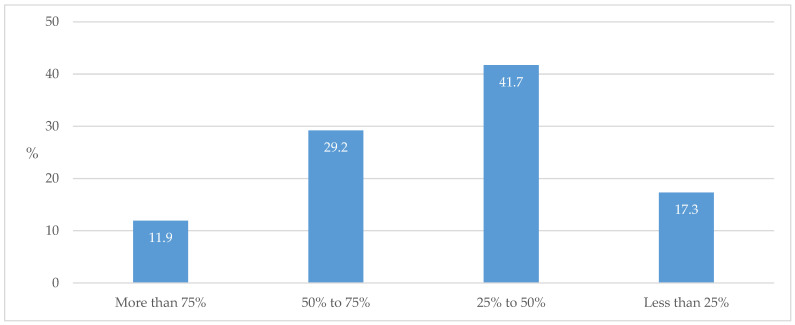
The proportion of specialist engagement in the treatment of individuals with T2D covered by this study. Respondents were asked to declare, based on their practice of referral to a specialist, to estimate the number of T2D cases for which they seek specialist consultation.

This study shows that more than half of individuals with T2D (59%) are solely under the care of a GP (Figure 6).

**Figure 7:**
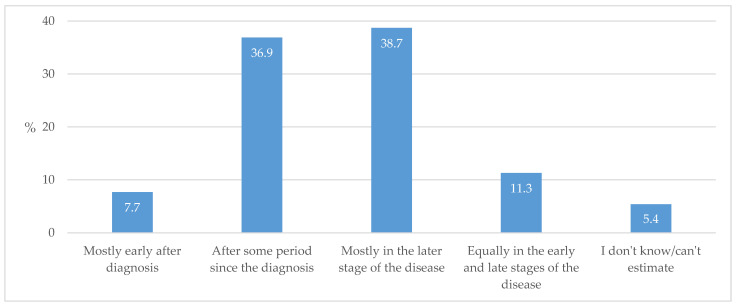
The stage of T2D when specialists were involved.

Regarding the stage of the disease when referral to a specialist most commonly occurred, the respondents most commonly declared referring after a certain period of duration of T2D (Figure 7).

**Figure 8:**
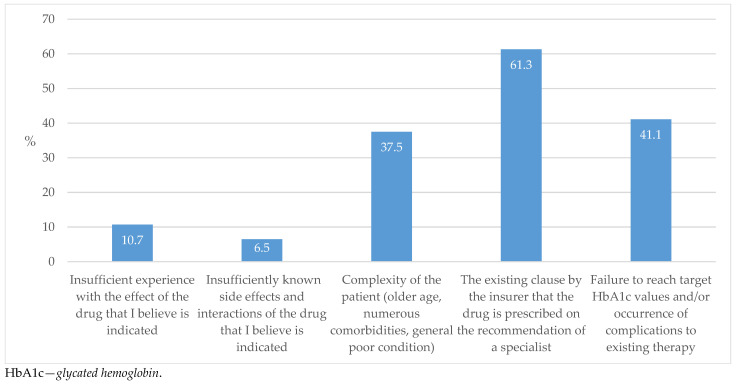
The most common priority indications for the referral of individuals with T2D to a specialist (Likert grade 5).

When asked about the most common reasons for a referral to a specialist, respondents dominantly highlighted a restrictive clause by insurers that prevents GPs from prescribing certain medications, failure to achieve targeted levels of HbA1c, and complexity of the patient (Figure 8). A detailed table with the frequencies of all the Likert grades can be found in Supplementary Table S4.

### 3.4. Quality of Care for T2D Individuals

**Figure 9:**
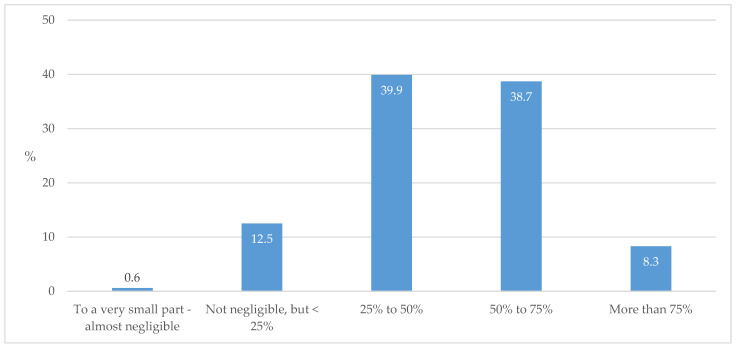
The proportion of individuals with well-controlled T2D in terms of achieving the target HbA1c level.

In this study, targeted levels of HbA1c, the most valuable marker for monitoring disease progression in individuals with diabetes, was reached in less than half of the T2D cases in the respondents’ practice (Figure 9).

**Figure 10:**
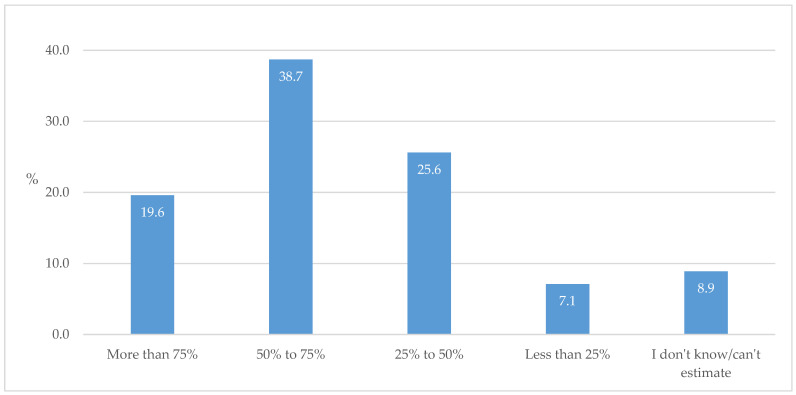
The proportion of simultaneously considering achieving targeted HbA1c levels and CV protection with cardio- and reno-protective medications in T2D cases.

The novel comprehensive approach with the dual principle of achieving targeted HbA1c levels and CV protection at the same time was applied in the treatment of around 60% of individuals with T2D (Figure 10).

**Figure 11:**
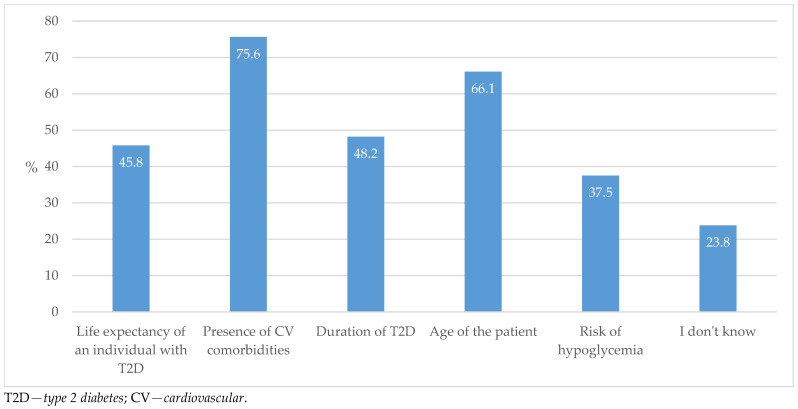
Factors used to determine satisfactory target values of HbA1c.

When it comes to determining satisfactory target values of HbA1c, the most selected factors influencing the decision making in this process were the presence of cardiovascular comorbidities and the age of the patient (Figure 11).

**Figure 12:**
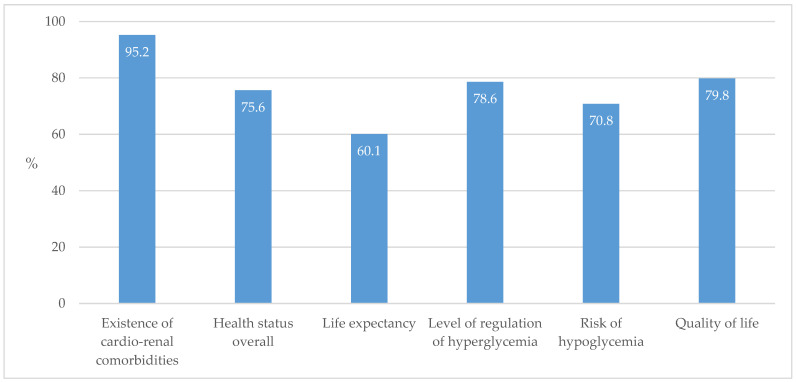
Factors that an individualized treatment approach for individuals with T2D should include.

While the respondents selected fairly equally all the provided factors in an individualized treatment approach to individuals with T2D, the existence of cardio-renal comorbidities stood out as the most selected factor (Figure 12).

### 3.5. GP’s Knowledge About Antidiabetic Medications

**Figure 13:**
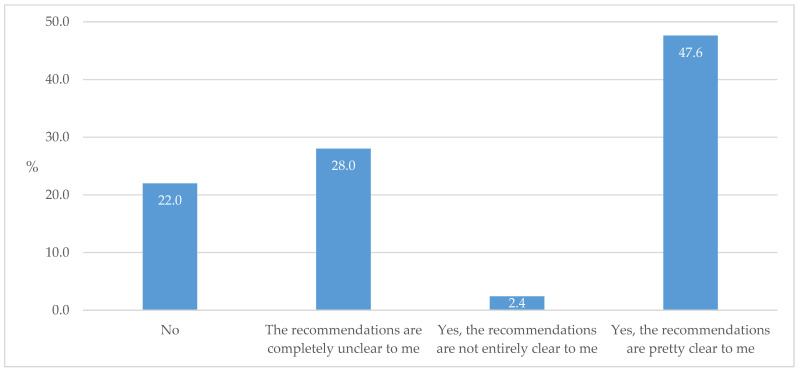
Familiarity with EASD/ADA guidelines for T2D treatment and for prescribing SGLT2ins and GLP-1 RAs.

Nearly two-thirds of the respondents claimed that they are familiar with the European Association for the Study of Diabetes (EASD)/ADA guidelines for the treatment of T2D. Still, slightly less than half of the respondents understood their recommendations completely clearly (Figure 13).

A good knowledge of T2D treatment guidelines and therapy options is crucial for ensuring quality care for individuals with diabetes; therefore, in Supplementary Table S5, we present an overview of the results related to the level of education and knowledge of the respondents.

### 3.6. Methods for Improving the Quality of Care for T2D Individuals

In order to optimize the prescription of antidiabetic medications with CV-protective effects and to improve the quality of care for individuals with T2D, the attitudes of clinicians and GPs are vastly important. In this regard, based on the positive models from the literature and everyday clinical practice, we offered several methods for improving the care of individuals with T2D. The respondents strongly agreed with all of the offered methods; however, those that were marked as most preferable (Likert grade 5) were implementing a structured display of data on individuals with T2D and incorporation of the exact algorithm of tests that need to be performed in an examination of individuals with T2D in their e-health profile (Table 5). A detailed list of Likert grades for all the methods can be found in Supplementary Table S6.

## 4. Discussion

This study is part of our larger research study aimed at examining practices of GPs in Croatia in prescribing antidiabetic medications, with an emphasis on cardio- and reno-protective medications. While in our previous paper we reported the results related to barriers that GPs encounter in the process of prescribing medications and their influence on GPs’ self-confidence [20], in this work, we focused primarily on the attitudes, habits, and practice of GPs in prescribing antidiabetic medications.

The importance of this study, as well as of related studies on the level of awareness of GPs about T2D treatment, stems from the fact that more than 53% of individuals with T2D in Croatia are solely under the care of GPs (Figure 6). If this is compared with other European countries, this proportion is even more pronounced in terms of the low number of specialist consultations [25,26]. It is an alarming fact that more than half of our respondents estimated that up to 50% of their patients do not reach the target HbA1c values (Figure 9); this seems to be a worldwide issue, as the multinational data are even worse, with 60% of those failing to reach the targeted goals [27]. The situation becomes even more severe when we consider the data indicating that most individuals with T2D have an associated CV comorbidity (Table 2). The question therefore arises: are the observed rates of prescribed cardio- and reno-protective medications too low?

Although the scientific field of research on novel antidiabetic medications is saturated, there are not yet many closely related studies worldwide to compare with. Still, there are several studies that have shown similar rates of prescription of these medications. A German study examined the rates of prescription of GLP-1 RAs and SGLT2ins after the implementation of EASD/ADA consensus guidelines in 2018 and declared similar findings to those obtained in our study [28]. While the prescription rates of GLP-1 RAs were up to 9.2% and those of SGLT2ins up to 20.4%, in our study, those rates were 14.4% and 20.9%, respectively (Table 2). Furthermore, one Swedish research claimed that only one-third of individuals with T2D who are suitable for treatment with SGLT2ins or GLP-1 Ras, according to EASD/ADA guidelines, have been prescribed these medications [29]. A recent real-life study conducted in Romania, aimed at assessing CV risk in individuals with T2D, found that SGLT2ins and GLP-1 RAs were prescribed in a total of 29.4% of T2D cases [30]. Even lower levels of prescribing of SGLT2ins and GLP-1 RAs were declared by a Spanish study, with proportion of 2.6% and 1.4%, respectively [31].

As stated, specialist diabetologists were involved in the process of treating individuals with T2D in less than half of cases. The primary reasons for referral to a specialist were the complexity of individuals with T2D, followed by failure to achieve target HbA1c values along with administrative restrictions in independently prescribing some medications. This primarily applies to GLP-1 RAs (Figure 8), as the prescribing of SGLT2ins in Croatia has been liberalized so that GPs can indicate their prescription on their own. This is not yet allowed for GLP-1 RAs in terms of treatment of T2D (allowed in obesity treatment), but GPs often express their initiative and readiness for doing so. In our previous paper, we reported that 76.2% of respondent GPs in Croatia declared a high level of self-confidence (Likert grades 4 and 5) to independently prescribe SGLT2ins and 53.6% in terms of GLP-1 RAs [20]. In comparison, French GPs seek specialist consultation predominantly due to the needs related to the introduction of insulin therapy and the consolidation of therapeutics and compliance, with an emphasis on advice for prescribing novel antidiabetic medications [25].

When searching for a reason for the lower prescription rates of GLP-1 RAs compared with SGLT2ins, administrative restrictions for prescribing GLP-1 RAs to individuals with T2D were imposed as a leading factor. Furthermore, as we reported in our recent paper, a certain number of respondents marked the injection form of a medication as a significant barrier when prescribing GLP-1 RAs due to patient refusal or decreased compliance [20]. As most of the GLP-1 RA medications have an injection form of application, and oral semaglutide came onto the Croatian market just a few years ago, these seem to be the main reasons for their lower prescription rates. In regard to the diversity of urban and rural areas of practices, there were higher prescription rates of novel antidiabetics in urban areas. Still, it remains unclear whether this is due to the greater awareness in GPs from urban areas or to the fact that in urban areas, a higher number of T2D individuals with associated CV comorbidities are also observed (Table 2).

The majority of respondents stated that they check and adjust medications for individuals with diabetes once a year or more often, which indicates a good process of managing and controlling (surveilling) these patients (Figure 3). In this case, medications are changed more frequently in individuals with a longer disease duration (Figure 4). However, in regard to the fact that specialist advice is equally sought after a shorter time from T2D diagnosis and after longer treatment (Figure 7), it is clear that uncertainty in prescribing medications arises early on in the initiation of treatment. The importance of timely and regular evaluations of T2D therapy has been shown by one recent systematic review that observed that individuals with T2D who did not achieve cardiometabolic control could achieve enhanced medication adherence and improvement in life quality by intensifying their GP encounter frequency [32].

If we consider the priorities by which GPs determine which HbA1c target values are satisfactory, they most often emphasize the presence of CV complications and the patient’s age, while the risk of hypoglycemia is less recognized as an important factor. About a quarter of respondents were not sure (did not know) which factors should be taken into account, which indicates insufficient knowledge (unfamiliarity with guidelines) and the need for better education (of international EASD/ADA and national guidelines) (Figure 11). This trend and the need for ongoing medical education is not surprising, as indicated by a very similar Belgian study, which determined that GPs lack optimal knowledge about the evidence-based use of SGLT2ins and even less knowledge with regard to GLP-1 RAs [33].

Furthermore, as a confusing factor in determining the clinical goals to be pursued during the long-term monitoring and surveillance of individuals with diabetes, the dual principle of determining treatment goals according to newer versions of the guidelines (simultaneous achievement of HbA1c target values and cardio- and reno-protection) should definitely be taken into account. More than half the GPs estimated that they consider the dual principle in over 50% of their patients (Figure 10). This indicates that a certain amount of time is needed to adapt to the new regime of management and supervision of individuals with T2D, which can be helped by continuous education and data from real-life studies, which can also help in creating more precise workflows and treatment protocols. One such study examined the impact of individualized HbA1c goals and patient awareness of their target values on medication adherence and achieved HbA1c values. Although achieving the HbA1c goal was not related to awareness of the goal, aware patients showed slightly more adherence to antidiabetic medications and tended to test their blood glucose levels more often [27].

The respondents answered that when prescribing antidiabetic medications, they mostly adhere to the principle of prescribing the medication with which they have the most experience in terms of its effect on lowering HbA1c, whose safety profile they are familiar with, and whose regimen of administration is simple for patients; these indicate a special model of working by GPs. Over time, they develop their own working style and decision-making system when prescribing medications to individual patients, where they rely mostly on their experience and adhere to the principle of primum non nocere while, at the same time, organizing the care and medication regimen according to the patient’s convenience and preferences (Supplementary Table S2). These data also reveal the deep causes of the inertia of GPs when new medications appear on the market, which could be reduced by introducing guidelines that would pay more attention to the indications of the medications and to the cost–benefit ratio for narrowly defined specific subgroups of patients. In this regard, guidelines specifically intended for general practice would be of great practical importance in terms of reducing inertia and optimizing the cost–benefit ratio in making decisions about prescribing medications. Precision medicine initiatives in diabetes that aim to identify subcategories of T2D patients using clustering techniques from data science applications to focus on CV risk and poor treatment outcomes could make a certain contribution [16].

Respondents also expressed their uncertainty about what the new guidelines recommend for specific patients, with the result indicating that most GPs prescribe fewer DPP-4ins, SGLT2ins, and GLP-1 RAs to older individuals with diabetes compared with to younger individuals (although the indications for these medications are more common in the elderly) (Supplementary Table S3). Accordingly, on average, only about one-third of individuals with T2D are prescribed medications with cardio- and reno-protective effects (Table 2). The established (and learned through regular education) caution of GPs in prescribing medications for the elderly, especially in terms of avoiding harmful interactions and polypharmacy, is also visible in the fact that in newly diagnosed cases, strategies of gradual medication introduction (one at a time) and immediate medication combinations (which would be according to indications for the prevention of CV complications and newer recommendations) are equally prevalent (Supplementary Table S3). This conflict is further clarified in the latest version of the EASD/ADA guidelines from 2024 [34].

The following data also show that this inert approach to the older age group of individuals with diabetes in relation to prescribing SGLT2ins and GLP-1 RAs is not a consequence of their deliberate neglect of the elderly due to the shortened life expectancy in this subgroup but rather a consequence of insufficient clarity about the effects of these medications in older individuals, their cost–benefit profile, as well as the fear of harming them with medications with which they personally do not have sufficient experience. In an individualized approach to treatment, GPs prioritized better quality over the assessment of life expectancy. Also, among the indicators of quality of care for individuals with diabetes, they recognized premature mortality in individuals with diabetes less than other indicators (Figure 12). In the context of the safety and necessity of applying SGLT2ins and GLP-1 RAs in older populations with T2D, apart from their well-known beneficial effects on cardiovascular and renal protection, a recent study brought new insights into their positive influence in reducing the risk of Alzheimer’s disease and related dementias [35].

Several results from this research study show that GPs in Croatia still adhere to the previously established ways of working and old paradigms but that they are slowly accepting new rules of care and recommendations for prescribing new, more effective medications. As leading indicators of quality of care and indications for prescribing novel antidiabetic medications, the GPs recognized old patterns in the form of target HbA1c values and the number of individuals with CV complications. When looking at the structure of these complications, the GPs recognized the importance of CAD more than peripheral vascular diseases (foot ulcers, leg amputations) and gave less importance to the reduction of renal function and heart failure as CV complications, while some other important comorbidities, such as acute urinary infections, were poorly recognized as indicators of quality of care for these patients (Table 3 and Supplementary Table S5). On the other hand, there is a tendency to reduce the share of traditional groups of antidiabetic medications prescribed, such as sulfonylurea medications (SFUs), with over 80% of the GPs claiming that they prescribe them in less than a quarter of their T2D cases (Supplementary Table S3), which correlates with the SFU rate of 19% observed from the Mediterranean population database [31]. This could be a significant area to work on in reducing clinical inertia.

Based on the answers by the GPs, it seems that the barriers to prescribing novel antidiabetic medications could be due more to unclear indications for their prescription and problems with accepting the dual principle of the treatment goal than to ignorance of the side effects of these medications. Therefore, most respondents rated the fear of side effects as a barrier to prescribing these medications as moderate (Likert grade 3) (Table 4). Still, in our previous paper, the results of multivariate regression analyses showed that familiarity with side effects reduces the probability of low self-confidence in the prescribing process, which indicates the vast significance of side effects [20].

Respondents were uncertain about the CV indications for prescribing GLP-1 RAs. The most common indications were achieving HbA1c target values and obesity, while the indications of ASCVD, such as CAD and especially other vascular complications, were less well recognized. Heart and kidney failure were mostly considered secondary indications (Likert grade 3), probably in accordance with the guidelines that SGLT2ins are to be recommended in the first line for these conditions. For milder degrees of heart failure (expressed as HLV), the indications were uncertain (Table 3). The implementation of periodic systematic cardiac ultrasounds, with exact data on the morphology and function of the heart, could help here.

Similarly, in the indications for prescribing SGLT2ins, GPs valued achieving the target HbA1c, which indicates the influence of the old paradigm in treatment but also the lack of clarity in the guidelines that state that these medications are the second line of choice in cases where target glycemic control is not achieved with metformin. Also, as in the case with GLP-1 RAs, CKD was less recognized as an important CV complication compared with CAD (which may be a consequence of long-term use in practice and the influence of algorithms for assessing CV risk in the general population). Similarly, the initial stages of heart failure were poorly recognized as an indication for SGLT2ins (Table 3). In addition to the implementation of systematic cardiac ultrasounds, learning from case reports and the implementation of online algorithms that help to determine the indications for pharmacological therapy according to the specifics of an individual patient would be possible solutions.

The most commonly prescribed first-line medications were primarily metformin, followed by metformin in combination with SGLT2ins (alone or in combination with metformin or DPP-4ins), which was the expected result according to current guidelines (Supplementary Table S3). The structure of antidiabetic medications prescriptions according to the importance of indications for clinical goals for CAD, CKD, and heart failure suggests that a transitional period is underway, with a shift from old to newer paradigms of care and groups of medications that are recommended (Appendix A, Table A1).

The most commonly prescribed medications for CAD were SGLT2ins (alone or in combination), while GLP-1 RAs (alone or in combination), DPP-4ins (alone or in combination), and the combination of SGLT2ins and GLP-1 RAs were less common and equally represented. Basal insulin was still highly represented among the prescribed medications, which also seems to be a practice of GPs in other countries [31]. The occupation of first place by SGLT2ins, instead of the expected GLP-1 RAs, may also reflect the restrictive prescription regime for GLP-1 RAs. In the structure of prescribing medications for CKD (eGFR < 60 mL), SGLT2ins (alone or in combination) were most commonly prescribed, followed equally by GLP-1 RAs (alone or in combination) and the combination of SGLT2ins and GLP-1 RAs, while basal insulin was still highly represented. This reflects the developmental stages in the appearance of medications on the market and the gradual transition by diabetologists, with recommendations for basal insulin in the case of emerging CV complications or advanced target organ damage and DPP-4ins (which are older-generation medications) or even GLP-1 RAs alone as well as the combination of SGLT2ins and GLP-1 RAs. In indications for heart failure, there was a tendency to reduce the share of SFUs and pioglitazone preparations as well as of metformin alone, while SGLT2ins were given priority (according to new recommendations, and GPs can also prescribe them independently), followed by GLP-1 RAs or combinations of SGLT2ins and GLP-1 RAs (Figure 5 and Table A1 (Appendix A)). Given the insights from similar data in related studies, Croatian GPs have a noticeably higher level of prescription of SGLT2ins and GLP-1 RAs in individuals with T2D and associated comorbidities [31].

The survey results show that there is still a need to work on better educating individuals with T2D, as well as on the education of GPs, as they were not sufficiently sure about which medications required dose adjustment in cases of reduced kidney function, about which antidiabetic medications could be combined, or about the impact of physical frailty on their effect. They showed a better knowledge of some issues, such as that contraindications for SGLT2ins include urinary tract infections and reduced kidney function of an eGFR < 45 mL, while they were poorly aware of incontinence and hypoglycemia. They also perceived well that the increased risk of developing CAD or CKD is most associated with a longer duration of diabetes and higher HbA1c values and that (but to a lesser extent) a younger age at diagnosis means an increased risk for these complications. However, they incorrectly perceived that men have a higher risk for these complications than women (which is probably influenced by the learned paradigm about the distribution of CV risk in the general population) (Supplementary Table S5). According to these results, it is necessary to focus the education of GPs on new data from evidence-based medicine and new data on antidiabetic medications.

As the respondents strongly concur with all of the offered methods for improving the care of individuals with T2D, it can be concluded that there is a wide field for the optimization and development of potential strategies for mitigating therapeutic inertia and the poor prescribing rates of novel antidiabetic medications. According to the GPs’ responses, it appears that the primary direction in which the evaluation of the above measures should be taken is the implementation of new aspects of monitoring individuals with diabetes and treatment algorithms based on up-to-date guidelines in patients’ e-health records (Table 5 and Supplementary Table S5). In this regard, considering the daily workload with a large number of patients and administration, e-health alerts such as alerts for CKD/HF patients to prompt SGLT2ins use would also be useful.

Finally, in terms of the treatment of individuals with obesity, GPs were cautious about introducing an injectable weight-loss medication (liraglutide), which more than half of respondents do not recommend yet. Those who did recommend it did so for individuals with obesity, with or without diabetes. They were not completely satisfied with this medication due to possible unwanted side effects (Supplementary Table S3).

A few elements can be determined as potential limitations of this study. The study obtained a slightly smaller number of participants than estimated; however, this should not have influenced the results as the sample thoroughly meets the representativeness of the national population of GPs and patients overall. Furthermore, in regard to the participant selection procedure, there might be a slight declination of the rules of randomization due to the specificities of the Croatian primary care system, which is divided into public and private practices. These declinations are related to the inability to reach all the GPs in the system due to unavailable e-mail addresses, and the fact that not all of the GPs are as yet adequately educated or equipped to access the e-survey. However, we consider that these limitations did not have an impact on the final sample, as the survey employed respondents from all the Croatian counties and age stratifications equally. Finally, registering exact numerical data about their patients by respondent GPs or according to their personal assessment can also be considered a potential limitation, because this may have had an impact on data accuracy.

Future research should change the design of studies on therapeutic inertia or the uptake of new medications. In addition to graphically displaying trends in the increase or decrease in the prescription of individual medication groups between two guideline updates, qualitative research should also be conducted on GPs’ attitudes to identify barriers that influence the development of therapeutic inertia. Also, they should detect and remediate gaps in GPs’ knowledge, using a case-study learning model from specific clinical situations. In addition to clinicians, professional associations, and scientists, the public health system also has an important task. In addition to increased work on educating the public about the prevention and treatment of T2D, the system should empower GPs and provide them with mechanisms for easier and briefer navigation and to set indications for prescribing medications through built-in treatment algorithms in their programs.

## 5. Conclusions

This study provides a comprehensive insight into the practice and attitudes of Croatian GPs in prescribing antidiabetic medications. It is an alarming fact that, according to our findings, over half of individuals with T2D are not well controlled in terms of achieving targeted HbA1c values. Furthermore, in terms of reducing their CV risk, although the data indicate a large proportion of associated comorbidities in individuals with T2D, low prescription rates of novel antidiabetic medications were observed. The results also indicate that in a large number of cases, Croatian GPs are still inclined to apply outdated paradigms of T2D treatment, which seems to be a consequence of a certain amount of resistance to change and of giving priority to established treatment patterns with which they already have some experience.

Finally, the large number of individuals with T2D who are solely under the care of GPs is the best indicator of the exceptional significance of such studies and of the need to work on optimizing the prescription of cardio- and reno-protective antidiabetic medications by GPs.

## Figures and Tables

**Table 1:** General characteristics of respondents (GPs).

Characteristics	N (%)
Total	168 (100.0)
**Gender**	
Male	57 (33.9)
Female	111 (66.1)
**Academic degree**	
Doctor without specialization	53 (31.5)
Family medicine resident	29 (17.3)
Family/general medicine specialist	83 (49.4)
Specialist in another field	3 (1.8)
**Place of practice**	
Urban area	114 (67.9)
Rural area	54 (32.1)

N—*number of respondents*.

**Table 2:** General characteristics of individuals with T2D in the study.

	N (%)			Median (IQR)
	Total	Urban Area	Rural Area	
**Individuals with T2D in the study**	23,036 (100.0)	16,511 (100.0)	6525 (100.0)	133 (95–179)
**<60 years** of age	6535 (28.4)	4638 (28.1)	1897 (29.1)	31 (20–50)
**60 to 80 years** of age	12,903 (56.0)	9409 (57.0)	3494 (53.5)	70 (50–102)
**>80 years** of age	3598 (15.6)	2464 (14.9)	1134 (17.4)	20 (10–30)
with associated **hypertension**	13,493 (58.6)	9812 (59.4)	3681 (56.4)	80 (50–100)
with associated **CAD**	4622 (20.0)	3473 (21.0)	1149 (17.6)	22 (10–40)
with associated **CKD (eGFR < 60 mL/min)**	4001 (17.4)	2836 (17.2)	1165 (17.9)	20 (10–34)
with associated **acute HF in the last year**	1164 (5.0)	882 (5.3)	282 (4.3)	4 (2–8)
who were prescribed **DPP-4ins**	8068 (35.0)	5907 (35.8)	2161 (33.1)	43 (30–64)
who were prescribed **SGLT2ins**	4828 (20.9)	3766 (22.8)	1062 (16.3)	25 (15–40)
who were prescribed **GLP-1 RAs**	3324 (14.4)	2522 (15.3)	802 (12.3)	15 (10–25)
who were prescribed **GLP-1 RAs** and **SGLT2ins combined**	1641 (7.1)	1234 (7.5)	407 (6.2)	6 (3–12)

N—*number of respondents*; IQR—*interquartile range*; T2D—*type 2 diabetes*; eGFR—*estimated glomerular filtration rate*; CAD—*coronary artery disease*; CKD—*chronic kidney disease*; HF—*heart failure*; DPP-4ins—*dipeptidyl peptidase-4 inhibitors*; GLP-1 RAs—*glucagon-like peptide-1 receptor agonists*; SGLT2ins—*sodium–glucose co-transporter 2 inhibitors*.

**Table 3:** Indications for prescribing SGLT2ins and GLP-1 RAs in individuals with T2D. Dark blue areas indicate more and light ones less frequently marked options.

	N (%)										
	The Lowest Priority		2		3		4		The Highest Priority		Total
Obesity	* 15 (9.2)	3 (1.8)	* 21 (12.9)	10 (6)	* 49 (30.1)	31 (18.6)	* 43 (26.4)	44 (26.3)	* 35 (21.5)	79 (47.3)	167 (100)
Achieving target HbA1c	0	1 (0.6)	5 (3)	7 (4.2)	23 (13.8)	32 (19.3)	54 (32.3)	53 (31.9)	85 (50.9)	73 (44)	166 (100)
CHD	1 (0.6)	9 (5.4)	8 (4.8)	17 (10.1)	22 (13.3)	32 (19)	61 (36.7)	58 (34.5)	74 (44.6)	52 (31)	168 (100)
HLV	2 (1.2)	13 (7.8)	16 (9.6)	26 (15.7)	36 (21.6)	53 (31.9)	61 (36.5)	50 (30.1)	52 (31.1)	24 (14.5)	166 (100)
Other vascular diseases **	1 (0.6)	10 (6)	19 (11.4)	21 (12.7)	40 (24.1)	43 (25.9)	57 (34.3)	62 (37.3)	49 (29.5)	30 (18.1)	166 (100)
CKD	2 (1.2)	10 (6)	10 (6)	22 (13.3)	27 (16.2)	39 (23.5)	48 (28.7)	60 (36.1)	80 (47.9)	35 (21.1)	166 (100)
Heart failure	0	8 (4.8)	10 (6)	18 (10.9)	21 (12.7)	45 (27.3)	41 (24.4)	50 (30.3)	96 (57.1)	44 (26.7)	165 (100)

* The column with data related to the prescription of SGLT2ins (otherwise, column contains data related to the prescription of GLP-1 RAs); N*—number of respondents*; HbA1c*—glycated hemoglobin*; CHD*—coronary heart disease*; HLV*—hypertrophy of the left ventricle*; ** cerebrovascular, peripheral arterial; CKD*—chronic kidney disease*.

**Table 4:** Fear of side effects as a barrier in the decision to prescribe antidiabetic medications.

	N (%)					
	The Least Important	2	3	4	The Most Important	Total
**DPP-4ins**	32 (19)	43 (25.6)	73 (43.5)	15 (8.9)	5 (3)	168 (100)
**GLP-1 RAs**	14 (8.3)	35 (20.8)	74 (44)	36 (21.4)	9 (5.4)	168 (100)
**SGLT2ins**	14 (8.3)	32 (19)	80 (47.6)	37 (22)	5 (3)	168 (100)

N—*number of respondents*; DPP-4ins—*dipeptidyl peptidase-4 inhibitors*; GLP-1 RAs—*glucagon-like peptide-1 receptor agonists*; SGLT2ins—*sodium–glucose cotransporter-2 inhibitors*.

**Table 5:** Selected methods for improving the care of individuals with T2D and for increasing the prescription of GLP-1 RAs and SGLT2ins, represented by the median of Likert grades 1 (strongly disagree) to 5 (strongly agree).

	Median (IQR)
Adding a panel in the e-health profile with indications for prescribing certain antidiabetic medications, their side effects, and a score for calculating the CV risk	4 (3–5)
Structured display of data on individuals with T2D in the e-health profile (all necessary data are systematically recorded for all patients)	5 (3–5)
Including questionnaires on the quality of life and cognitive dysfunction, daily activity performance, and the presence of physical weakness syndrome in the patient’s e-health profile	4 (3–4)
Adding a system for monitoring care quality indicators for individuals with T2D to the e-health profile	4 (3–4)
Education aimed at improving doctor–patient communication so that the circumstances of the patient’s life, cognitive abilities, experience with previous therapy, level of health literacy, and personal preferences for a certain medication or method of medication administration are considered	4 (3–5)
Installation of an online proxy to help decide on a certain form of therapy, with alternative therapeutic options	4 (3–5)
Incorporation of the exact algormitm of tests in e-health profile that need to be performed in an individual with T2D, especially including a reminder for the analysis of renal function and for referring for ECGs, carotid ultrasounds, and dynamic ECGs	5 (3–5)
At the local level: the establishment of an expert interdisciplinary group that develops guidelines and writes them in a simpler and more understandable form so that GPs can easily apply them	4 (3–5)
Learning from patient examples	4 (3–5)
The decision to prescribe these medications should be left entirely to GPs without restrictions on their prescription by a specialist	3 (3–4)
Greater interdisciplinarity in the organization of health care for individuals with T2D, e.g., through the establishment of a dispensary or a good functional connection between professions	4 (3–5)
Education on the pharmaco-economics for GPs, internists, and the legislature, which would increase awareness of the profitability of using medications that may be more expensive in price but that are ultimately cheaper for the health system, considering their effectiveness in reducing CV outcomes	4 (3–5)
Organizing a continuing education course on the results of CVOTs, which could increase GPs’ awareness of the importance of the CV risk, and not just of hyperglycemia, in the treatment of T2D	4 (3–5)
Issuance of information leaflets for patients on the possibility of an increased CV risk if they do not take new cardio- and reno-protective medications and the risk of side effects if they take them	3 (3–5)
None of that would be effective; the most important thing when prescribing medications is my experience and my personal assessment of the patient’s suitability for a particular therapy option	2 (1–3)

IQR—*interquartile range*; CV—*cardiovascular*; T2D—*type 2 diabetes*; ECG—*electrocardiogram*; GPs—*general practitioners*; CVOTs—*cardiovascular outcome trials*.

## Data Availability

This comprehensive research yielded a wealth of data, which will be published in several publications. To enhance the understanding of the collected data and to clarify the content of the study, all the data generated or analyzed in this part of the research are included in this published article and in Appendix A.

## Supplementary Materials

The following supporting information can be downloaded at: https://www.mdpi.com/article/10.3390/biomedicines13061491/s1, Table S1: Regional diversity of respondents and individuals under their care; Table S2: Factors contributing in the decision of medication prescription in individuals with T2D; Table S3: The habits and attitudes of respondents in the practice of prescribing antidiabetic medications; Table S4: Indications for referral of individuals with T2D to a specialist; Table S5: GPs’ knowledge about treatment of T2D and complications; Table S6: Methods for improving the quality of care of individuals with T2D; Table S7: STROBE Statement—Checklist of items that should be included in reports of cross-sectional studies.

## Abbreviations

The following abbreviations are used in this manuscript:

T2D	type 2 diabetes
CV	cardiovascular
HF	heart failure
CVD	cardiovascular disease
FM	family medicine
DPP-4ins	dipeptidyl peptidase-4 inhibitors
HbA1c	glycated hemoglobin
ADA	American Diabetes Association
ASCVD	atherosclerotic cardiovascular disease
CKD	chronic kidney disease
SGLT2ins	sodium–glucose cotransporter-2 inhibitors
GLP-1 RAs	glucagon-like peptide-1 receptor agonists
CVOTs	cardiovascular outcome trials
GPs	general practitioners
CAD	coronary artery disease
eGFR	estimated glomerular filtration rate
HLV	hypertrophy of the left ventricle
EASD	European Association for the Study of Diabetes
ECG	electrocardiogram
SFUs	sulfonylurea medications

## Appendix A

**Table A1:** Medications prescribed to individuals with T2D and their associated comorbidity.

	N (%)			
	The Least Prescribed	2	The Most Prescribed	Total
Individuals with T2D and **CAD**				
Metformin	57 (42.5)	47 (35.1)	30 (22.4)	134 (100)
DPP-4ins	36 (24.7)	70 (47.9)	40 (27.4)	146 (100)
GLP-1 RAs	29 (20.3)	69 (48.3)	45 (31.5)	143 (100)
SGLT2ins	8 (4.9)	57 (35.2)	97 (59.9)	162 (100)
Both SGLT2ins and GLP-1 RAs	36 (25.7)	61 (43.6)	43 (30.7)	140 (100)
Sulfonylurea	100 (75.8)	25 (18.9)	7 (5.3)	132 (100)
Pioglitazone	83 (64.8)	37 (28.9)	8 (6.3)	128 (100)
Basal insulin	36 (26.3)	71 (51.8)	30 (21.9)	137 (100)
Individuals with T2D and **CKD (eGFR < 60 mL/min)**				
Metformin	72 (52.2)	39 (28.3)	27 (19.6)	138 (100)
DPP-4ins	34 (23.9)	70 (49.3)	38 (26.8)	142 (100)
GLP-1 RAs	34 (23.4)	80 (55.2)	31 (21.4)	145 (100)
SGLT2ins	12 (7.7)	74 (47.4)	70 (44.9)	156 (100)
Both SGLT2ins and GLP-1 RAs	40 (28.2)	63 (44.4)	39 (27.5)	142 (100)
Sulfonylurea	85 (65.4)	37 (28.5)	8 (6.2)	130 (100)
Pioglitazone	83 (63.8)	38 (29.2)	9 (6.9)	130 (100)
Basal insulin	38 (27.7)	63 (46)	36 (26.3)	137 (100)
Individuals with T2D and **HF**				
Metformin	73 (54.1)	38 (28.1)	24 (17.8)	135 (100)
DPP-4ins	49 (35)	65 (46.4)	26 (18.6)	140 (100)
GLP-1 RAs	35 (24.5)	77 (53.8)	31 (21.7)	143 (100)
SGLT2ins	10 (6.2)	48 (29.8)	103 (64)	161 (100)
Both SGLT2ins and GLP-1 RAs	36 (25.5)	61 (43.3)	44 (31.2)	141 (100)
Sulfonylurea	94 (74)	27 (21.3)	6 (4.7)	127 (100)
Pioglitazone	89 (71.2)	28 (22.4)	8 (6.4)	125 (100)
Basal insulin	50 (37.6)	62 (46.6)	21 (15.8)	133 (100)

N—*number of respondents*; CAD—*coronary artery disease*; DPP-4ins—*dipeptidyl peptidase-4 inhibitors*; GLP-1 RAs—*glucagon-like peptide-1 receptor agonists*; SGLT2ins—*sodium–glucose cotransporter-2 inhibitors*; T2D—*type 2 diabetes*; CKD—*chronic kidney disease*; eGFR—*estimated glomerular filtration rate*; HF—*heart failure*.
