# Supplementary material for: Prescribing Antidiabetic Medications Among GPs in Croatia—A Real-Life Cross-Sectional Study
Source: Biomedicines. 2025 Jun 17;13(6):1491. doi: 10.3390/biomedicines13061491 (PMC12190737; doi:10.3390/biomedicines13061491)
Supplement: Supplementary file 1 [file biomedicines-13-01491-s001.zip › biomedicines-3645699-supplementary.pdf]

Table S1. Regional diversity of the respondents and the number of individuals under their care.

	N(%)		
	Respondents	Individuals under the care	Individuals with T2D
**Total**	**168 (100.0)**	**263,806 (100.0)**	**23,036 (8.7)**
**City of Zagreb**	**18 (10.7)**	**31,732 (12)**	**2,487 (7.8)**
**Northern Croatia**	**17 (10.1)**	**28,244 (10.7)**	**2,271 (8)**
Krapina–Zagorje County	5 (3)	7,055 (2.7)	520 (7.4)
Međimurje County	3 (1.8)	6,325 (2.4)	457 (7.2)
Varaždin County	3 (1.8)	5,448 (2.1)	527 (9.7)
Koprivnica–Križevci County	6 (3.6)	9,416 (3.6)	767 (8.1)
**Central Croatia**	**18 (10.7)**	**28,841 (10.9)**	**2,640 (9.1)**
Zagreb County	6 (3.6)	9,856 (3.7)	909 (9.2)
Sisak–Moslavina County	5 (3)	8,255 (3.1)	774 (9.4)
Karlovac County	2 (1.2)	2,227 (0.8)	269 (12.1)
Bjelovar–Bilogora County	3 (1.8)	5,430 (2.1)	499 (9.2)
Lika–Senj County	2 (1.2)	3,073 (1.2)	189 (6.2)
**Eastern Croatia**	**59 (35.1)**	**88,882 (33.7)**	**7,472 (8.4)**
Osijek–Baranja County	35 (20.8)	50,396 (19.1)	4,382 (8.7)
Virovitica–Podravina County	3 (1.8)	4,765 (1.8)	450 (9.4)
Vukovar–Srijem County	12 (7.1)	17,946 (6.8)	1,510 (8.4)
Požega–Slavonia County	0	0	0
Brod–Posavina County	9 (5.4)	15,775 (6)	1,130 (7.2)
**Western Croatia**	**27 (16)**	**41,046 (15.6)**	**4,024 (9.8)**
Istria County	10 (6)	16,669 (6.3)	1,492 (9)
Primorje–Gorski Kotar County	17 (10.1)	24,377 (9.2)	2,532 (10.4)
**Southern Croatia**	**29 (17.3)**	**45,061 (17)**	**3,842 (8.5)**
Zadar County	5 (3)	9,035 (3.4)	320 (6.9)
Šibenik–Knin County	6 (3.6)	8,292 (3.1)	897 (10.8)
Split–Dalmatia County	16 (9.5)	24,044 (9.1)	2,100 (8.7)
Dubrovnik–Neretva County	2 (1.2)	3,690 (1.4)	525 (14.2)

N—*number of respondents*; T2D—*type 2 diabetes*.

Table S2. Factors contributing to the decision to prescribe medication to individuals with T2D.

	N (%)					
	**The least important**	**2**	**3**	**4**	**The most important**	**Total**
According to the guidelines, the effect of this medication is clearly described	2 (1.2)	7 (4.2)	36 (21.4)	58 (34.5)	65 (38.7)	168 (100)
A medication with which I have a lot of experience in terms of lowering HbA1c	0	4 (2.4)	22 (13.1)	71 (42.3)	71 (42.3)	168 (100)
I know the safety profile of the medication (side effects, interactions)	0	2 (1.2)	21 (12.5)	60 (35.7)	85 (50.6)	168 (100)
The administration regimen is simple for patients	1 (0.6)	3 (1.8)	25 (14.9)	64 (38.1)	75 (44.6)	168 (100)
Knowing for sure which patients it is suitable for (taking into account indications, side effects, the patient's health condition and life expectancy)	0	3 (1.8)	30 (17.9)	63 (37.5)	72 (42.9)	168 (100)
Are the prescribing restrictions (contraindications) clearly defined for that medication according to the guidelines?	0	6 (3.6)	27 (16.1)	64 (38.1)	71 (42.3)	168 (100)
The price of the medication and the amount of co-payment for patients	5 (3)	11 (6.5)	41 (24.4)	60 (35.7)	51 (30.4)	168 (100)
Should the clause of the insurer be fulfilled (prescription according to the specialist's recommendation)?	7 (4.2)	9 (5.4)	31 (18.5)	44 (26.2)	77 (45.8)	168 (100)

N—*number of respondents*; HbA1c—*glycated hemoglobin*.

Table S3. The habits and attitudes of respondents in the practice of prescribing antidiabetic medications.

	N (%)	
Do you prescribe certain medications to elderly people (> 65 years)?	DPP-4ins	SGLT2ins
Rarely – almost not at all	14 (8.3)	24 (14.3)
Yes, but less than for people younger than that age	119 (70.8)	98 (58.3)
Yes, more than for people younger than that age	35 (20.8)	46 (27.4)
What is your usual treatment pattern for a newly diagnosed individual with T2D aged over 65 years?		
Gradual introduction of oral antidiabetic medications—one by one		74 (44)
I immediately introduce two medications or a combination of medications		79 (47)
I have no opinion on that		5 (3)
I believe that basal insulin is needed		5 (3)
In that case, I refer the patient to a diabetologist to decide what is best for that patient		5 (3)
The proportion of individuals with T2D prescribed SFUs in your practice		
To a very small part—almost negligible		72 (42.9)
Not negligible but < 25%		68 (40.5)
25% to 50%		24 (14.3)
50% to 75%		4 (2.4)
Which of these medications are prescribed more often than others as the first-line choice in individuals with T2D in your practice?		
Metformin alone		103 (61.3)
SFUs alone		3 (1.8)
DPP-4ins alone		1 (0.6)
Pioglitazone alone		5 (3)
GLP-1 RAs alone		25 (14.9)
Combinations of metformin and SFUs or DPP-4ins		2 (1.2)
Combinations of pioglitazone and SFUs or DPP-4ins		25 (14.9)
SGLT2ins (alone or in combination with metformin or DPP-4ins)		4 (2.4)
In the treatment of newly diagnosed T2D and in the presence of one of the cardiovascular complications*, I would prescribe GLP-1 RAs or SGLT2ins as the first option of therapy		130 (77.4)
Have you ever recommended liraglutide for weight loss to your patients?		
Yes, for individuals with obesity and T2D		34 (20.2)
Yes, for individuals with obesity but without diabetes		7 (4.2)
Yes, for individuals with obesity with and without diabetes		38 (22.6)
No, I have not		89 (53)
How satisfied are you with the effect of liraglutide?		
I am not satisfied with its effect on weight loss and/or blood glucose regulation		10 (6)
I have noticed side effects that may affect the prescription of this medication		8 (4.8)
I am satisfied with its effect, but not with the side effects (safety profile)		34 (20.2)
I am completely satisfied with the effectiveness and safety profile of this medication		27 (16.1)

N—*number of respondents*; DPP-4ins—*dipeptidyl peptidase-4 inhibitors*; SGLT2ins—*sodium–glucose cotransporter-2 inhibitors*; T2D—*type 2 diabetes*; SFUs—*sulfonylurea medications*; GLP-1 Ras—*glucagon-like peptide-1 receptor agonists*; **myocardial infarction, cerebrovascular disease, peripheral arterial disease or foot ulcers, heart failure, left heart ventricule hypertrophy, microalbuminuria, renal failure.*

Table S4. Indications for referral of individuals with T2D to a specialist.

	N (%)					
	**The lowest priority**	**2**	**3**	**4**	**The highest priority**	**Total**
Insufficient experience with the effect of the medication that I believe is indicated	40 (23.8)	43 (25.6)	40 (23.8)	27 (16.1)	18 (10.7)	168 (100)
Insufficiently known side effects and interactions of the medication that I believe is indicated	43 (25.6)	44 (26.2)	49 (29.2)	21 (12.5)	11 (6.5)	168 (100)
Complexity of the patient (older age, numerous comorbidities, general poor condition)	6 (3.6)	13 (7.7)	35 (20.8)	51 (30.4)	63 (37.5)	168 (100)
The clause by the insurer that the medication is prescribed only on the recommendation of a specialist	4 (2.4)	7 (4.2)	19 (11.3)	35 (20.8)	103 (61.3)	168 (100)
Failure to reach target HbA1c values and/or occurrence of complications with existing therapy	2 (1.2)	7 (4.2)	33 (19.6)	57 (33.9)	69 (41.1)	168 (100)

N—*number of respondents*; HbA1c—*glycated hemoglobin*.

Table S5. GPs' knowledge about the treatment of T2D and its complications.

	N (%)
They have heard of CVOTs that demonstrate better CV outcomes if GLP-1 RAs and SGLT2ins are prescribed to individuals with T2D	156 (92.9)
Factors that increase the risk of developing CAD in individuals with T2D	
The longer the duration of T2D	140 (83.3)
The younger the patient is at the time of diagnosis of T2D	97 (57.7)
The older the patient is at the time of diagnosis with T2D	31 (18.5)
The higher the HbA1c	137 (81.5)
If the patient is male	24 (14.3)
If the patient is female	85 (50.6)
Factors that increase the risk of developing renal failure in individuals with T2D	
The longer the duration of T2D	146 (86.9)
The younger the patient is at the time of diagnosis of T2D	91 (54.2)
The older the patient is at the time of diagnosis with T2D	42 (25.0)
The higher the HbA1c	140 (83.3)
If the patient is male	24 (14.3)
If the patient is female	55 (32.7)
For which of the medications do you have to watch out for reduced renal function?	
Empaglifosine	72 (42.9)
Liraglutide	47 (28.0)
Semaglutide	46 (27.4)
Sitagliptin	63 (37.5)
Saxagliptin	57 (33.9)
None	40 (23.8)
Which medicines can be taken together?	
DPP-4ins and GLP-1 RAs	21 (12.5)
DPP-4ins and SGLT2ins	54 (32.1)
GLP-1 RAs and SGLT2ins	66 (39.3)
All combinations are possible	86 (51.2)
Neither combination can be prescribed	17 (10.1)
Does the state of frailty change the effect of antidiabetic medications?	
I have not heard of that condition	37 (22)
Yes, it does change	74 (44)
No, it does not change	10 (6)
I do not know	47 (28)
What are the limitations when prescribing SGLT2ins to individuals with T2D?	
Decreased renal function (eGFR<60 mL/min)	20 (11.9)
Decreased renal function (eGFR<45 mL/min)	85 (50.6)
Tendency to hypoglycemia	18 (10.7)
Urinary incontinence	52 (31)
Chronic (recurrent) infections of the urinary tract	123 (73.2)
Reduced life expectancy	1 (0.6)
I do not know	13 (7.7)

N—*number of respondents*; CVOTs—*cardiovascular outcome trials;* CV—*cardiovascular*; SGLT2ins—*sodium–glucose cotransporter-2 inhibitors*; GLP-1 Ras—*glucagon-like peptide-1 receptor agonists*; T2D—*type 2 diabetes*; CAD—*coronary artery disease*; HbA1c—*glycated hemoglobin*; DPP-4ins—*dipeptidyl peptidase-4 inhibitors*; eGFR – *estimated glomerular filtration rate*.

Table S6. Methods for improving the quality of care of individuals with T2D.

	N (%)					
	**The least benefit**	**2**	**3**	**4**	**The greatest benefit**	**Total**
Adding a panel in the e-health profile with indications for prescribing certain antidiabetics, their side effects, and a score for calculating the CV risk	7 (4.2)	21 (12.5)	34 (20.2)	44 (26.2)	62 (36.9)	168 (100)
Structured display of data on individuals with T2D in the e-health profile (all necessary data are systematically recorded for all patients)	4 (2.4)	10 (6)	36 (21.4)	57 (33.9)	61 (36.3)	168 (100)
Including questionnaires on the quality of life and cognitive dysfunction, daily activity performance, and the presence of physical weakness syndrome in the patient's e-health profile	15 (8.9)	24 (14.3)	40 (23.8)	56 (33.3)	33 (19.6)	168 (100)
Adding a system to the e-health profile for monitoring care quality indicators for individuals with T2D	8 (4.8)	16 (9.5)	54 (32.1)	53 (31.5)	37 (22)	168 (100)
Education aimed at improving doctor–patient communication so that the circumstances of the patient's life, cognitive abilities, experience with previous therapy, level of health literacy, and personal preferences for a certain medication or method of medication administration are considered	6 (3.6)	16 (9.5)	37 (22)	55 (32.7)	54 (32.1)	168 (100)
Installation of an online proxy to help decide on a certain form of therapy, with alternative therapeutic options	9 (5.4)	10 (6)	48 (28.6)	51 (30.4)	50 (29.8)	168 (100)
Incorporation of the exact algorithm of tests in the e-health profile that need to be performed in an individual with T2D, especially including a reminder for the analysis of renal function and for referring for ECGs, carotid ultrasounds, and dynamic ECGs	5 (3)	10 (6)	43 (25.6)	39 (23.2)	71 (42.3)	168 (100)
At the local level: the establishment of an expert interdisciplinary group that develops guidelines and writes them in a simpler and more understandable form so that GPs can easily apply them	11 (6.5)	15 (8.9)	50 (29.8)	41 (24.4)	51 (30.4)	168 (100)
Learning from patient examples	0	15 (8.9)	55 (32.7)	49 (29.2)	49 (29.2)	168 (100)
The decision to prescribe these medications should be left entirely to GPs without restrictions on their prescription by a specialist	8 (4.8)	25 (14.9)	63 (37.5)	32 (19)	40 (23.8)	168 (100)
Greater interdisciplinarity in the organization of health care for individuals with T2D, e.g., through the establishment of a dispensary or a good functional connection between professions	9 (5.4)	16 (9.5)	46 (27.4)	52 (31)	45 (26.8)	168 (100)
Education on pharmaco-economics for GPs, internists and the legislature, which would increase awareness of the profitability of using medications that may be more expensive in price but that are ultimately cheaper for the health system considering their effectiveness in reducing CV outcomes	10 (6)	18 (10.7)	44 (26.2)	44 (26.2)	52 (31)	168 (100)
Organizing a continuing education course on the results of CVOTs, which could increase GPs' awareness of the importance of the CV risk, and not just hyperglycemia, in the treatment of T2D	8 (4.8)	5 (3)	52 (31)	51 (30.4)	52 (31)	168 (100)
Issuance of information leaflets for patients on the possibility of increased CV risk if they do not take new cardio- and reno-protective medications and the risk of side effects if they take them	16 (9.5)	18 (10.7)	50 (29.8)	40 (23.8)	44 (26.2)	168 (100)
None of that would be effective; the most important thing when prescribing medications is my experience and my personal assessment of the patient's suitability for a particular therapy option	63 (37.5)	35 (20.8)	45 (26.8)	15 (8.9)	10 (6)	168 (100)

N—*number of respondents*; CV—*cardiovascular*; T2D—*type 2 diabetes*; ECG—*electrocardiogram*; GPs—*general practitioners*; CVOTs—*cardiovascular outcome trials*.

Table S7. STROBE Statement—Checklist of items that should be included in reports of cross-sectional studies

	Item No	Recommendation	Present in study
**Title and abstract**	1	(*a*) Indicate the study's design with a commonly used term in the title or the abstract.	Yes
		(*b*) Provide in the abstract an informative and balanced summary of what was done and what was found.	Yes
**Introduction**			
Background/rationale	2	Explain the scientific background and rationale for the investigation being reported.	Yes
Objectives	3	State specific objectives, including any prespecified hypotheses.	Yes
**Methods**			
Study design	4	Present key elements of the study design early in the paper.	Yes
Setting	5	Describe the setting, locations, and relevant dates, including periods of recruitment, exposure, follow-up, and data collection.	Yes
Participants	6	Give the eligibility criteria and the sources and methods of selection of participants.	Yes
Variables	7	Clearly define all outcomes, exposures, predictors, potential confounders, and effect modifiers. Give diagnostic criteria, if applicable.	Yes
Data sources/ measurement	8*	For each variable of interest, give sources of data and details of methods of assessment (measurement). Describe the comparability of assessment methods if there is more than one group.	Yes
Bias	9	Describe any efforts to address potential sources of bias.	Yes
Study size	10	Explain how the study size was arrived at.	Yes
Quantitative variables	11	Explain how quantitative variables were handled in the analyses. If applicable, describe which groupings were chosen and why.	Yes
Statistical methods	12	(*a*) Describe all statistical methods, including those used to control for confounding.	Yes
		(*b*) Describe any methods used to examine subgroups and interactions.	Yes
		(*c*) Explain how missing data were addressed.	N/A
		(*d*) If applicable, describe the analytical methods, taking into account the sampling strategy.	N/A
		(*e*) Describe any sensitivity analyses.	N/A
**Results**			
Participants	13*	(*a*) Report the numbers of individuals at each stage of study—e.g., numbers of those potentially eligible, examined for eligibility, confirmed eligible, included in the study, completing follow-up, and analysed.	Yes
		(*b*) Give reasons for non-participation at each stage.	Yes
		(*c*) Consider the use of a flow diagram.	Yes
Descriptive data	14*	(*a*) Give characteristics of study participants (e.g., demographic, clinical, social) and information on exposures and potential confounders.	Yes
		(*b*) Indicate the number of participants with missing data for each variable of interest.	N/A
Outcome data	15*	Report numbers of outcome events or summary measures.	Yes
Main results	16	(*a*) Provide unadjusted estimates and, if applicable, confounder-adjusted estimates and their precision (e.g., 95% confidence interval). Make clear which confounders were adjusted for and why they were included.	Yes

		(*b*) Report category boundaries when continuous variables are categorized.	Yes
		(*c*) If relevant, consider translating estimates of relative risk into values of absolute risk for a meaningful time period.	No
Other analyses	17	Report other analyses performed—e.g., analyses of subgroups and interactions and sensitivity analyses.	No
**Discussion**			
Key results	18	Summarize key results with reference to the study objectives.	Yes
Limitations	19	Discuss limitations of the study, taking into account sources of potential bias or imprecision. Discuss both the direction and magnitude of any potential bias.	Yes
Interpretation	20	Give a cautious overall interpretation of the results, considering the objectives, limitations, multiplicity of analyses, results from similar studies, and other relevant evidence.	Yes
Generalizability	21	Discuss the generalizability (external validity) of the study results.	Yes
**Other information**			
Funding	22	Give the source of funding and the role of the funders for the present study and, if applicable, for the original study on which the present article is based.	Yes

*Give information separately for exposed and unexposed groups.

Note: An Explanation and Elaboration article discusses each checklist item and provides the methodological background and published examples of transparent reporting. The STROBE checklist is best used in conjunction with this article (freely available on the websites of PLoS Medicine at http://www.plosmedicine.org/, Annals of Internal Medicine at http://www.annals.org/, and Epidemiology at http://www.epidem.com/). Information on the STROBE Initiative is available at www.strobe-statement.org.
